# Supplementary material for: Transcriptomic Profiling of Bronchial Epithelium Reveals Dysregulated Interferon and Inflammatory Responses to Rhinovirus in Exacerbation-Prone Pediatric Asthma
Source: bioRxiv. 2025 Oct 19:2025.10.19.683298. Preprint. [Version 1] doi: 10.1101/2025.10.19.683298 (PMC12633054; doi:10.1101/2025.10.19.683298)
Supplement: 1 [file NIHPP2025.10.19.683298V1-supplement-1.pdf]

bioRxiv preprint doi: https://doi.org/10.1101/2025.10.19.683298; this version posted October 19, 2025. The copyright holder for this preprint (which was not certified by peer review) is the author/funder, who has granted bioRxiv a license to display the preprint in perpetuity. It is made available under aCC-BY 4.0 International license.

Supplemental material

Supplemental Figure 1. Organotypic bronchial epithelial cultures. (**A**) Representative hematoxylin and eosin (H&E) stained histology cross sections and (**B**) scanning electron microscopy images of passage 3 primary bronchial epithelial cultures from study asthma donors differentiated to an organotypic pseudostratified and ciliated state for 21 days under air-liquid interface conditions using PneumaCult™ALI medium (Stemcell™).

bioRxiv preprint doi: https://doi.org/10.1101/2025.10.19.683298; this version posted October 19, 2025. The copyright holder for this preprint (which was not certified by peer review) is the author/funder, who has granted bioRxiv a license to display the preprint in perpetuity. It is made available under aCC-BY 4.0 International license.

Supplemental Figure 2. Time course of Module Expression and Correlation with Viral Load, with Functional Enrichment Annotations (**A, B**) Scatterplots showing the non-linear changes in expression of the "Interferon Response" module over time by donor group. BECs from SE Asthma donors exhibited a significantly higher average expression level at 2 days, with a 1.69-fold increase compared to NSE Asthma donors and a 1.92-fold increase compared to Healthy donors with a significantly different GAMM shape fit characterized by sustained higher levels at each subsequent timepoint (SE vs NSE: GAMM All timepoint: FDR=3.48e-05, linear model effect size restricted to d2: Estimate=0.76, FDR=9.47e-03, SE vs Healthy: GAMM All timepoint: FDR=9.87e-07, linear model effect size restricted to d2: Estimate=0.94, FDR=1.35e-1). (**C**) Selected enriched GO, Biocarta, Reactome, MSigDB Hallmark and KEGG terms for the 363 genes within the "Interferon Response" module. Dot size denotes the k/K value (ratio of number of genes in module (k) divided by the number of genes in the indicated geneset (K)), and the color denotes statistical significance of the pathway enrichment at FDR ranging from 0-0.20. (**D, E**) Analogous plots of the "Epithelial Remodeling and Inflammation" module. BECs from SE Asthma donors demonstrated a 1.15-fold higher expression level at 2d compared to NSE donors and sustained elevation at 4d with return to baseline levels in the NSE asthma group (SE vs NSE: GAMM All timepoint: FDR=8.4e-03, linear model effect size restricted to d2: Estimate=0.20, FDR=6.50e-01), and SE Asthma donors demonstrated a sustained greater expression compared to Healthy donors at each timepoint with a 1.67-fold higher expression at 2d (SE vs Healthy: GAMM All timepoint: FDR=6.55e-03, linear model effect size restricted to d2: Estimate=0.74, FDR=2.87e-01). (**F, G**) Selected enriched GO, Biocarta, Reactome, MSigDB Hallmark and KEGG terms for the 1396 genes within the "Epithelial Remodeling and Inflammation" module. Dot size denotes the k/K value (ratio of number of genes in module (k) divided by the number of genes in the indicated geneset (K)), and the color denotes statistical significance of the pathway enrichment at FDR ranging from 0-0.20. (**H, I**) Analogous plots of the "Stress Response" module. BECs from SE Asthma donors demonstrated a 1.23-fold higher

bioRxiv preprint doi: https://doi.org/10.1101/2025.10.19.683298; this version posted October 19, 2025. The copyright holder for this preprint (which was not certified by peer review) is the author/funder, who has granted bioRxiv a license to display the preprint in perpetuity. It is made available under aCC-BY 4.0 International license.

expression level at 2d compared to NSE donors and a 1.33-fold higher expression compared to Healthy donors, with sustained higher levels over time (SE vs NSE: GAMM All timepoint: FDR=1.8e-3, linear model effect size restricted to d2: Estimate=0.30, FDR=1.66e-02, SE vs Healthy: GAMM All timepoint: FDR=7.73e-04, linear model effect size restricted to d2: Estimate=0.41, FDR=1.53e-01). (**J**) Selected enriched GO, Biocarta, Reactome, MSigDB Hallmark and KEGG terms for the 242 genes within the “Stress Response" module. Dot size denotes the k/K value (ratio of number of genes in module (k) divided by the number of genes in the indicated geneset (K)), and the color denotes statistical significance of the pathway enrichment at FDR ranging from 0-0.20. (**K, L**) Scatterplot showing the non-linear changes in expression of the “Cellular Metabolism” module over time differing by clinical exacerbation group. BECs from SE Asthma donors demonstrated a significantly lower average expression level at 2 days, with a 1.30-fold decrease compared to NSE Asthma donors and a 1.52-fold decrease compared to Healthy donors with a significantly different GAMM shape fit characterized by sustained lower levels at each subsequent timepoint (SE vs NSE: GAMM All timepoint:FDR=8.1e-04, linear model effect size restricted to d2: Estimate=-0.38, FDR=1.66e-02, SE vs Healthy: GAMM All timepoint: FDR=9.80e-05, linear model effect size restricted to d2: Estimate=-0.60, FDR=1.12e-01). (**M**) Selected enriched GO, Biocarta, Reactome, MSigDB Hallmark and KEGG terms for the 106 genes within the “Cellular Metabolism" module. Dot size denotes the k/K value (ratio of number of genes in module (k) divided by the number of genes in the indicated geneset (K)), and the color denotes statistical significance of the pathway enrichment at FDR ranging from 0-0.20. (**N, O**) Analogous plots of the “Cellular Transcriptional Activity” module. BECs from SE donors demonstrated a significantly lower average expression level at 2 days, with a 1.21-fold decrease compared to NSE donors and a 1.32-fold decrease compared to Healthy donors with a significantly different GAMM shape fit characterized by sustained lower levels at each subsequent timepoint (SE vs NSE: GAMM All timepoint:FDR =6.8e-04, linear model effect size restricted to d2: Estimate=-0.28, FDR=1.66e-02, SE vs

bioRxiv preprint doi: https://doi.org/10.1101/2025.10.19.683298; this version posted October 19, 2025. The copyright holder for this preprint (which was not certified by peer review) is the author/funder, who has granted bioRxiv a license to display the preprint in perpetuity. It is made available under aCC-BY 4.0 International license.

Healthy: GAMM All timepoint: FDR=2.40e-03, linear model effect size restricted to d2: Estimate=-0.41, FDR=1.35e-01). (**P**) Selected enriched GO, Biocarta, Reactome, MSigDB Hallmark and KEGG terms for the 204 genes within the “Cellular Transcriptional Activity" module. Dot size denotes the k/K value (ratio of number of genes in module (k) divided by the number of genes in the indicated geneset (K)), and the color denotes statistical significance of the pathway enrichment at FDR ranging from 0-0.20.

bioRxiv preprint doi: https://doi.org/10.1101/2025.10.19.683298; this version posted October 19, 2025. The copyright holder for this preprint (which was not certified by peer review) is the author/funder, who has granted bioRxiv a license to display the preprint in perpetuity. It is made available under aCC-BY 4.0 International license.

Supplemental Figure 3. Viral load has a mediating effect on Module expression. A linear model subset to the post infection timepoints, d2-10, was used to assess the extent to which viral load differences were mediating the magnitude differences in module expression observed between SE and NSE groups. Four of the modules of interest, Interferon response, Stress response, Cellular metabolism, and Cellular transcriptional activity, showed that viral load mediated the majority of difference in module expression observed between the two groups. The differences between SE and NSE in the Interferon response module were: without viral load: Estimate=0.42, p=9.56e-03; with viral load: Estimate=-0.045, p=0.69. Stress response module: without viral load: Estimate=0.14, p=0.024; with viral load: Estimate= -0.0014, p=0.98. Cellular metabolism module: without viral load: Estimate=-0.13, p=0.058; with viral load: Estimate=0.024, p=0.66. Cellular transcriptional activity module: without viral load: estimate=-0.13, p=0.028; with viral load: Estimate=0.0033, p=0.95. No result could be derived for the Epithelial Remodeling and Inflammation module as this module largely differed by kinetic shape between groups rather than overall magnitude of expression Shown are the model estimates on x-axis and p-values on the y-axis for the model without (purple) or with (pink) viral load included in the model. The 5 modules of interest are labeled.

bioRxiv preprint doi: https://doi.org/10.1101/2025.10.19.683298; this version posted October 19, 2025. The copyright holder for this preprint (which was not certified by peer review) is the author/funder, who has granted bioRxiv a license to display the preprint in perpetuity. It is made available under aCC-BY 4.0 International license.

Supplemental Figure 4. Post-Infection Viral load shows a significant inverse relationship to Pre-Infection CXCL10. Scatter plot showing a significant inverse relationship between log transformed pre-infection CXCL10 protein secretion and log transformed viral load post-infection d2-d10.

bioRxiv preprint doi: https://doi.org/10.1101/2025.10.19.683298; this version posted October 19, 2025. The copyright holder for this preprint (which was not certified by peer review) is the author/funder, who has granted bioRxiv a license to display the preprint in perpetuity. It is made available under aCC-BY 4.0 International license.

Supplemental Figure 5. Pre-Infection CXCL10 shows a significant inverse relationship to Day 2 CXCL10, IFNβ, IFN-λ2/IL-28A and IFN-λ3/IL-28B. Scatter plot showing a significant inverse relationship between log transformed pre-infection CXCL10 protein secretion and subsequent protein secretion at 2 days post RV infection of (**A**) CXCL10 (Estimate=-0.37, p=7.16e-03), (**B**) IFN-λ2 (Estimate=-0.69, p=2.32e-05), and, (**C**) IFN-λ3 (Estimate=-0.34, p=4.24e-03) as well as a non-significant inverse trend with (**D**) IFN-β (Estimate=-0.14, p=1.69e-01). Fit lines are based on a linear model including 95% confidence intervals.

bioRxiv preprint doi: https://doi.org/10.1101/2025.10.19.683298; this version posted October 19, 2025. The copyright holder for this preprint (which was not certified by peer review) is the author/funder, who has granted bioRxiv a license to display the preprint in perpetuity. It is made available under aCC-BY 4.0 International license.

Supplemental Figure 6. Module Expression Difference by RV16, RV16 + IFN-β and RV16 + CXCL10 (**A**) Dot plot showing expression changes in Interferon response module by IFN-β treatment showing a 1.53-fold decrease in expression with IFN-β treatment compared to RV16 (estimate=-0.6, p=1.23e-03) and whereas there is a 4.42-fold and a 2.88-fold increase in expression from Pre-infection compared to RV16 infected sample and IFN-β treated samples respectively (RV16:estimate=2.14, p=2.74e-15, IFN-β:estimate=1.52, p=4.25e-11). (**B**) Dot plot showing expression changes in Epithelial remodelling and Inflammation response module by IFN-β treatment showing a 1.20-fold decrease in expression with IFN-β treatment (estimate=-0.27, p=2.24e-03) and whereas there is a 1.47-fold and a 1.21-fold increase in expression from Pre-infection compared to RV16 infected sample and IFN-β treated samples respectively (RV16:estimate=0.55, p=9.41e-09, IFN-β:estimate=0.28, p=3.23e-04). (**C**) Dot plot showing expression changes in Stress response module by IFN-β treatment showing a 1.14-fold decrease in expression with IFN-β treatment (estimate=-0.19, p=4.61e-03) and whereas there is a 1.35-fold and a 1.18-fold increase in expression from Pre-infection compared to RV16 infected sample and IFN-β treated samples respectively (RV16:estimate=0.43, p=1.39e-08, IFN-β:estimate=0.23, p=2.41e-04). (**D**) Dot plot showing expression changes in Cellular metabolism module by IFN-β treatment showing a 1.12-fold increase in expression with IFN-β treatment (estimate=0.16, p=4.20e-02) and whereas there is a 1.48-fold and a 1.32-fold decrease in expression from Pre-infection compared to RV16 infected sample and IFN-β treated samples respectively (RV16:estimate=-0.56, p=1.18e-08, IFN-β:estimate=-0.40, p=5.64e-06). (**E**) Dot plot showing expression changes in Cellular transcriptional activity module by IFN-β treatment showing a 1.09-fold increase in expression with IFN-β treatment (estimate=0.13, p=2.13e-02) and whereas there is a 1.31-fold and a 1.19-fold decrease in expression from Pre-infection compared to RV16 infected sample and IFN-β treated samples respectively (RV16:estimate=-0.39, p=1.18e-08, IFN-β:estimate=-0.25, p=1.57e-05) . Boxplots indicate median and

bioRxiv preprint doi: https://doi.org/10.1101/2025.10.19.683298; this version posted October 19, 2025. The copyright holder for this preprint (which was not certified by peer review) is the author/funder, who has granted bioRxiv a license to display the preprint in perpetuity. It is made available under aCC-BY 4.0 International license.

interquartile ranges for each group and the Fit line is based on a linear model including 95% confidence intervals.

bioRxiv preprint doi: https://doi.org/10.1101/2025.10.19.683298; this version posted October 19, 2025. The copyright holder for this preprint (which was not certified by peer review) is the author/funder, who has granted bioRxiv a license to display the preprint in perpetuity. It is made available under aCC-BY 4.0 International license.

Supplemental Figure 7. Single cell transcriptomics, module level analysis. (**A**) UMAP representation of the 316,712 cells separated by donor and condition (uninfected and RV infection). (**B**) Heatmap showing relative expression of modules (defined by WGCNA from bulk transcriptomics) expression at 48 hr following RV infection across donor groups, healthy, non-Severe and Severe at individual cell types . Module expression levels are shown as row normalized Z-scores of the mean expression for each group with red representing higher relative expression and blue representing lower relative expression. Module row ordering is by hierarchical clustering. (**C**) Violin plot showing distribution of module expression for select modules by condition (uninfected and RV infection) by donor groups, healthy, non-Severe and Severe at individual cell types. The black horizontal line in each violin denotes a mean expression.

bioRxiv preprint doi: https://doi.org/10.1101/2025.10.19.683298; this version posted October 19, 2025. The copyright holder for this preprint (which was not certified by peer review) is the author/funder, who has granted bioRxiv a license to display the preprint in perpetuity. It is made available under aCC-BY 4.0 International license.

Supplemental Figure 8. Single cell transcriptomics, gene level analysis. (**A**) Upset plot showing the count of overlapping genes with descending trend across five cell types, basal, secretory, goblet, ciliated and secretory immune response cells. (**B**) Heatmap showing relative expression of 48 core signature genes (shared across all five cell types) with descending expression trends at 5 cell types. Gene expression levels are shown as row normalized Z-scores of the mean expression for each group with red representing higher relative expression and blue representing lower relative expression.

bioRxiv preprint doi: https://doi.org/10.1101/2025.10.19.683298; this version posted October 19, 2025. The copyright holder for this preprint (which was not certified by peer review) is the author/funder, who has granted bioRxiv a license to display the preprint in perpetuity. It is made available under aCC-BY 4.0 International license.

Supplemental Figure 1

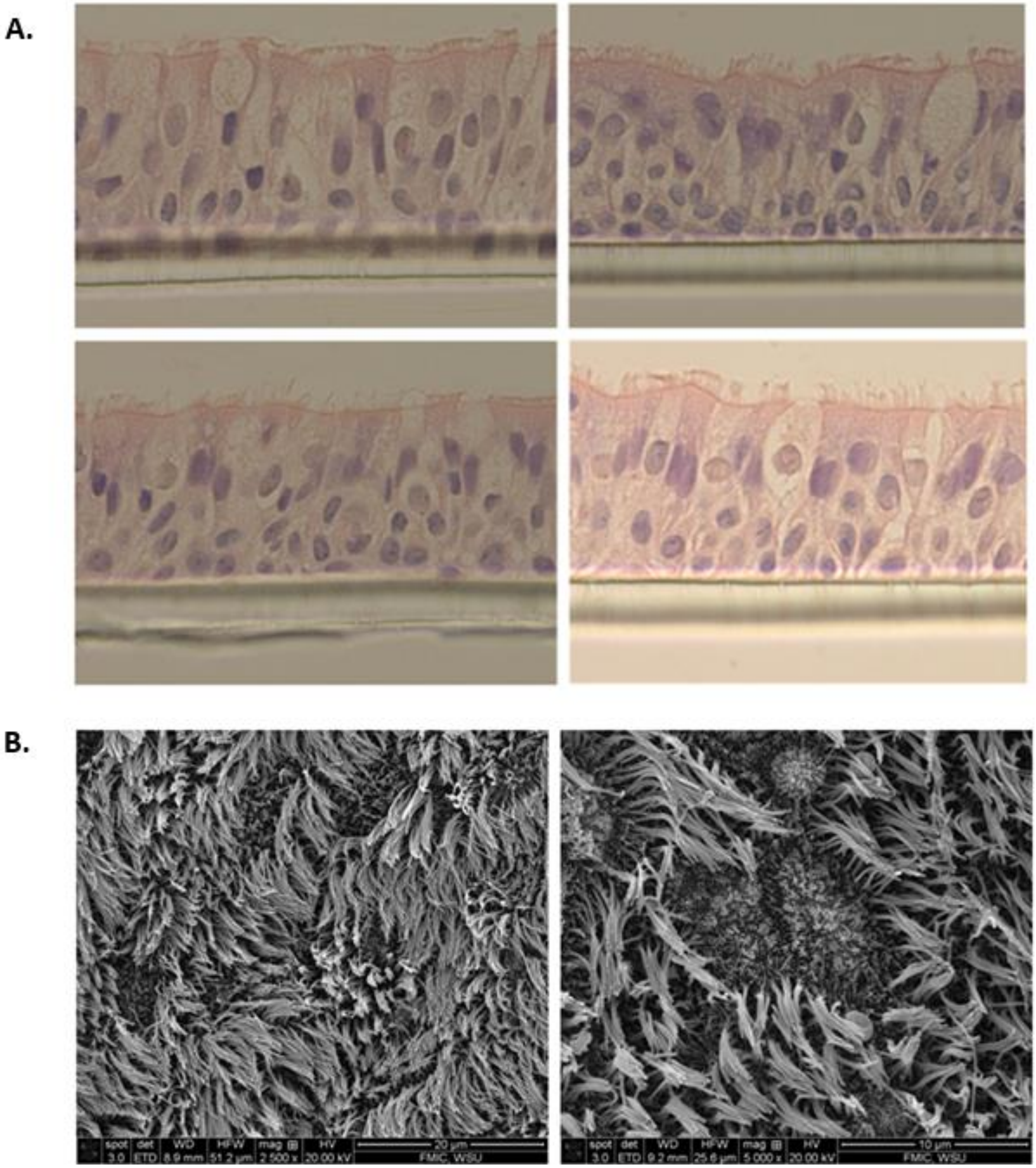

bioRxiv preprint doi: https://doi.org/10.1101/2025.10.19.683298; this version posted October 19, 2025. The copyright holder for this preprint (which was not certified by peer review) is the author/funder, who has granted bioRxiv a license to display the preprint in perpetuity. It is made available under aCC-BY 4.0 International license.

Supplemental Figure 2

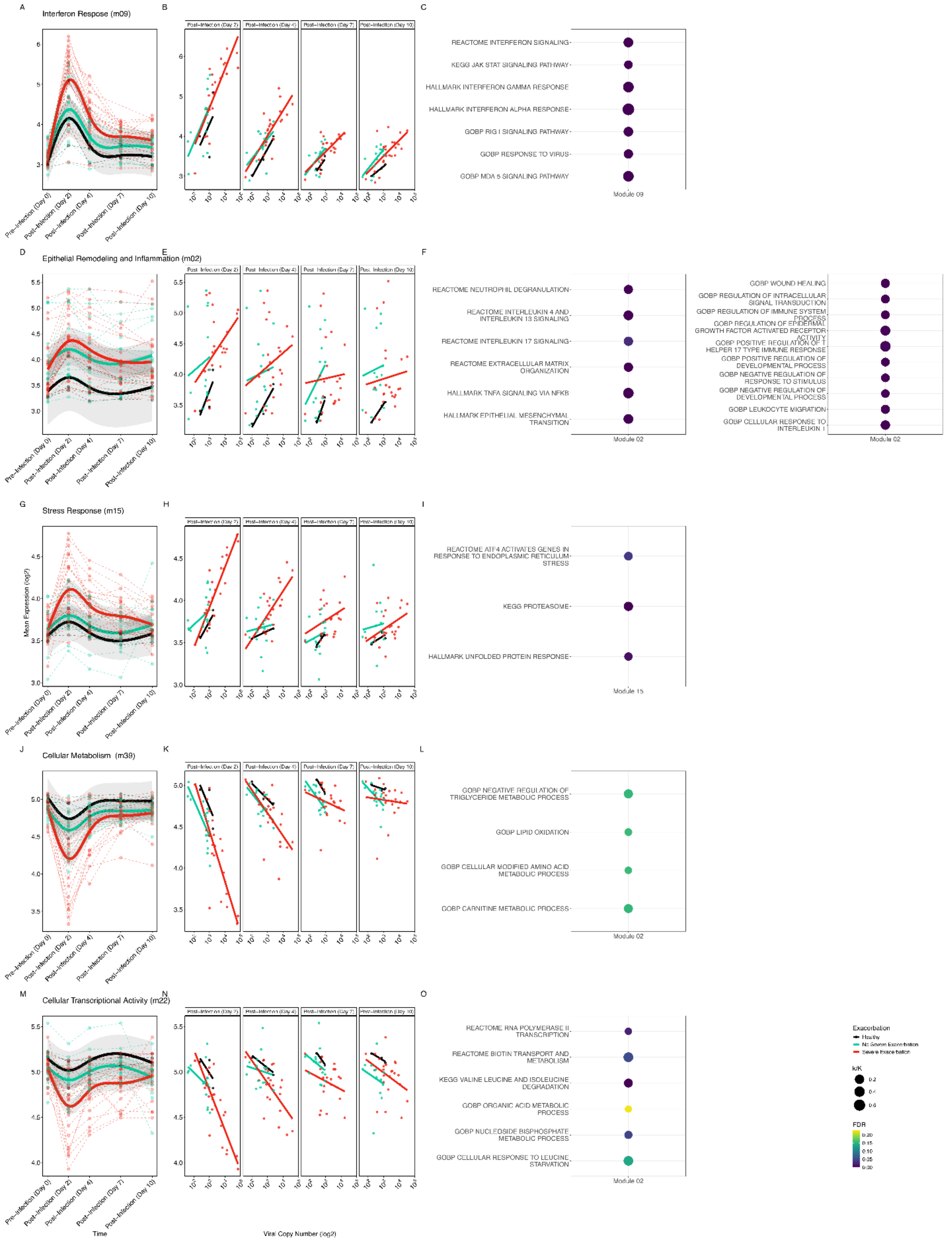

bioRxiv preprint doi: https://doi.org/10.1101/2025.10.19.683298; this version posted October 19, 2025. The copyright holder for this preprint (which was not certified by peer review) is the author/funder, who has granted bioRxiv a license to display the preprint in perpetuity. It is made available under aCC-BY 4.0 International license.

Supplemental Figure 3

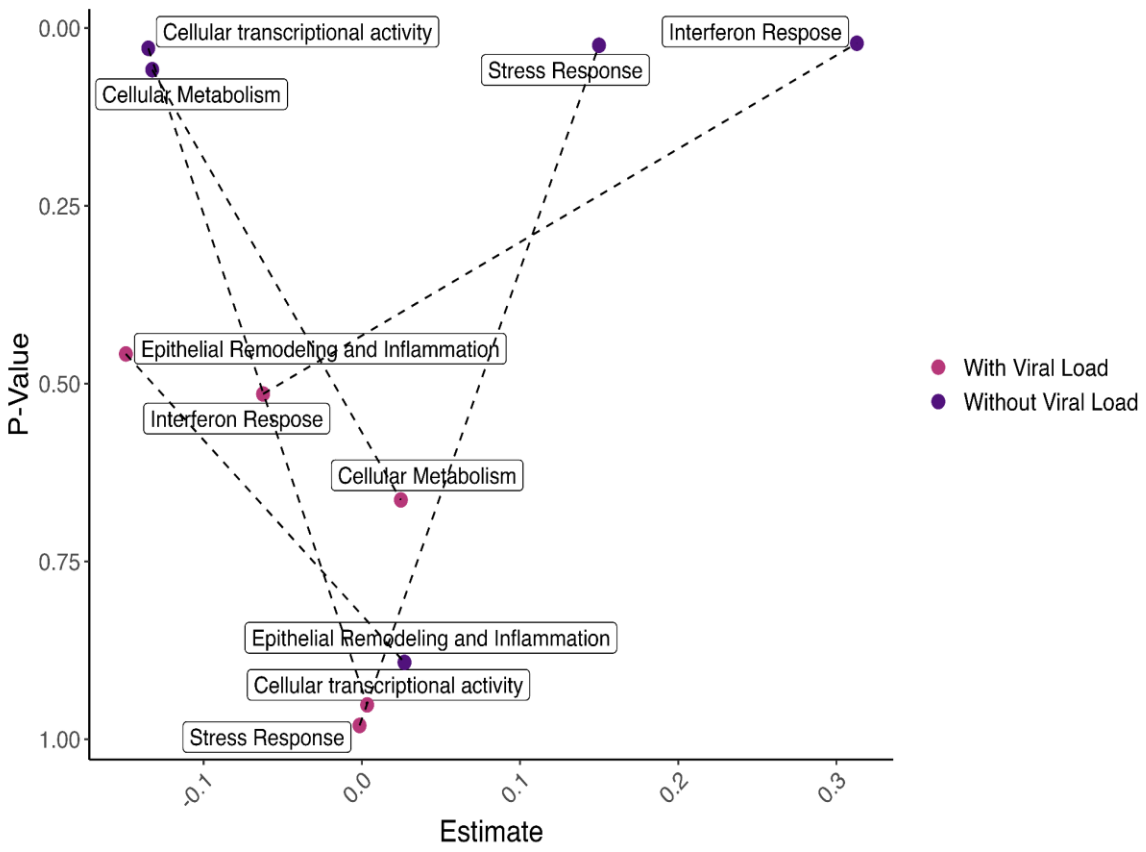

bioRxiv preprint doi: https://doi.org/10.1101/2025.10.19.683298; this version posted October 19, 2025. The copyright holder for this preprint (which was not certified by peer review) is the author/funder, who has granted bioRxiv a license to display the preprint in perpetuity. It is made available under aCC-BY 4.0 International license.

Supplemental Figure 4

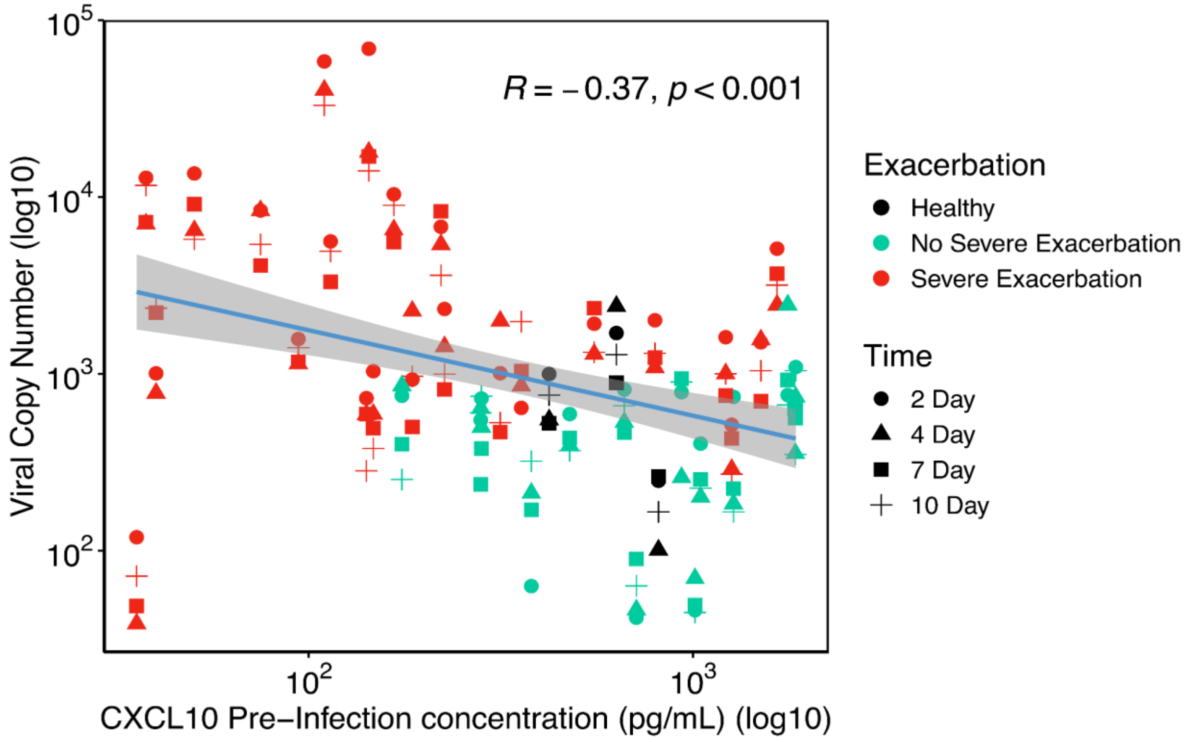

bioRxiv preprint doi: https://doi.org/10.1101/2025.10.19.683298; this version posted October 19, 2025. The copyright holder for this preprint (which was not certified by peer review) is the author/funder, who has granted bioRxiv a license to display the preprint in perpetuity. It is made available under aCC-BY 4.0 International license.

Supplemental Figure 5

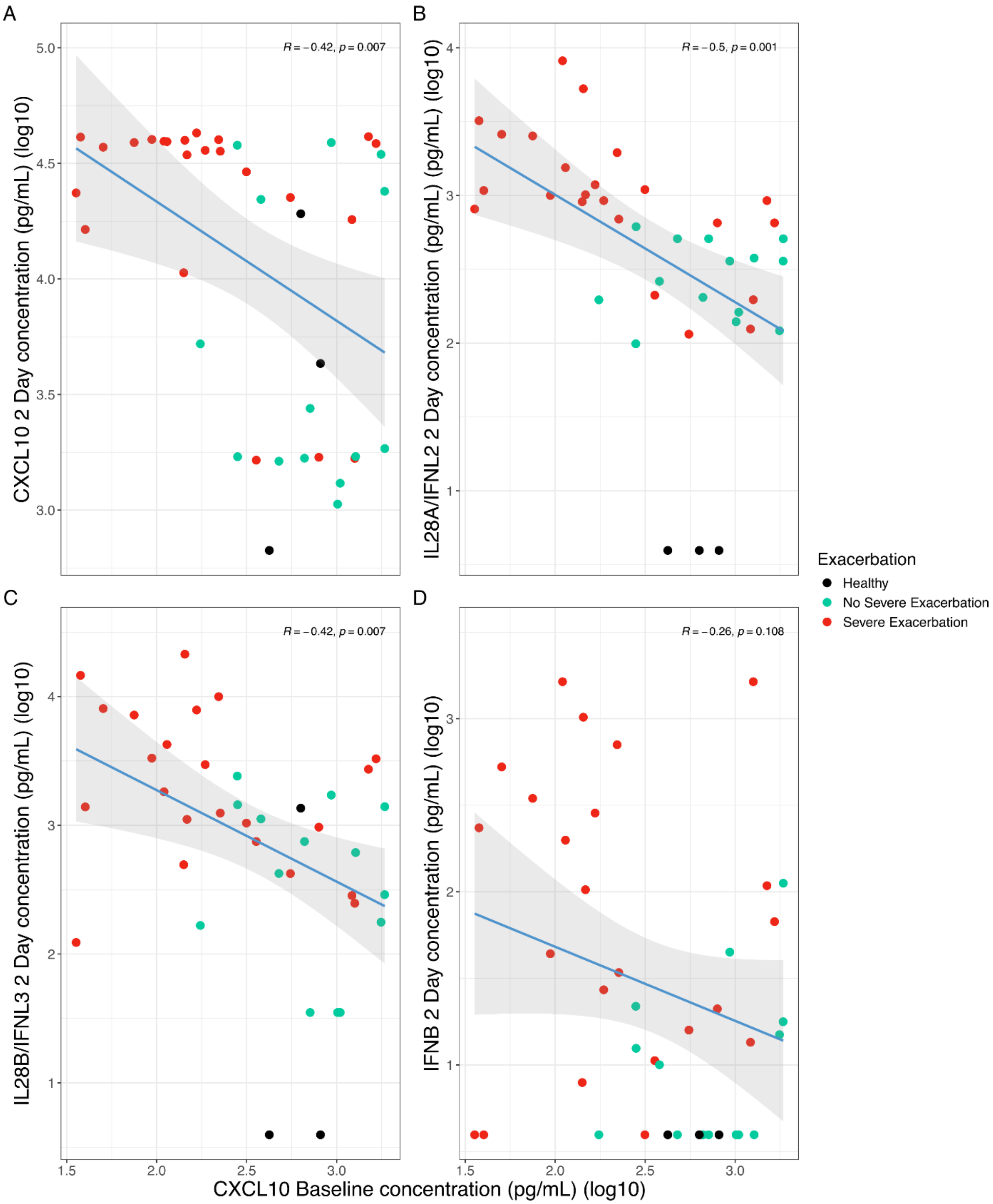

bioRxiv preprint doi: https://doi.org/10.1101/2025.10.19.683298; this version posted October 19, 2025. The copyright holder for this preprint (which was not certified by peer review) is the author/funder, who has granted bioRxiv a license to display the preprint in perpetuity. It is made available under aCC-BY 4.0 International license.

Supplemental Figure 6

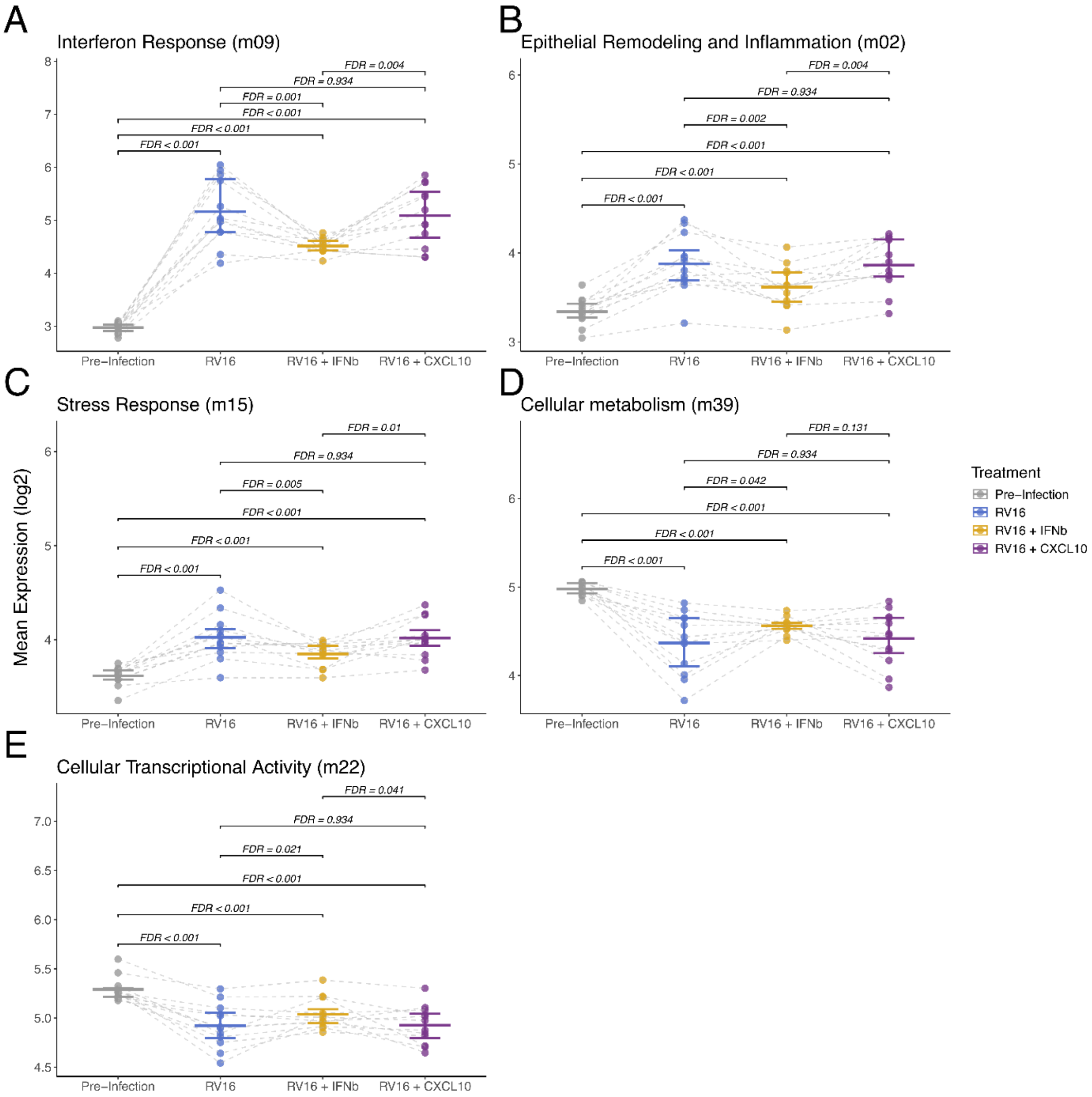

bioRxiv preprint doi: https://doi.org/10.1101/2025.10.19.683298; this version posted October 19, 2025. The copyright holder for this preprint (which was not certified by peer review) is the author/funder, who has granted bioRxiv a license to display the preprint in perpetuity. It is made available under aCC-BY 4.0 International license.

Supplemental Figure 7

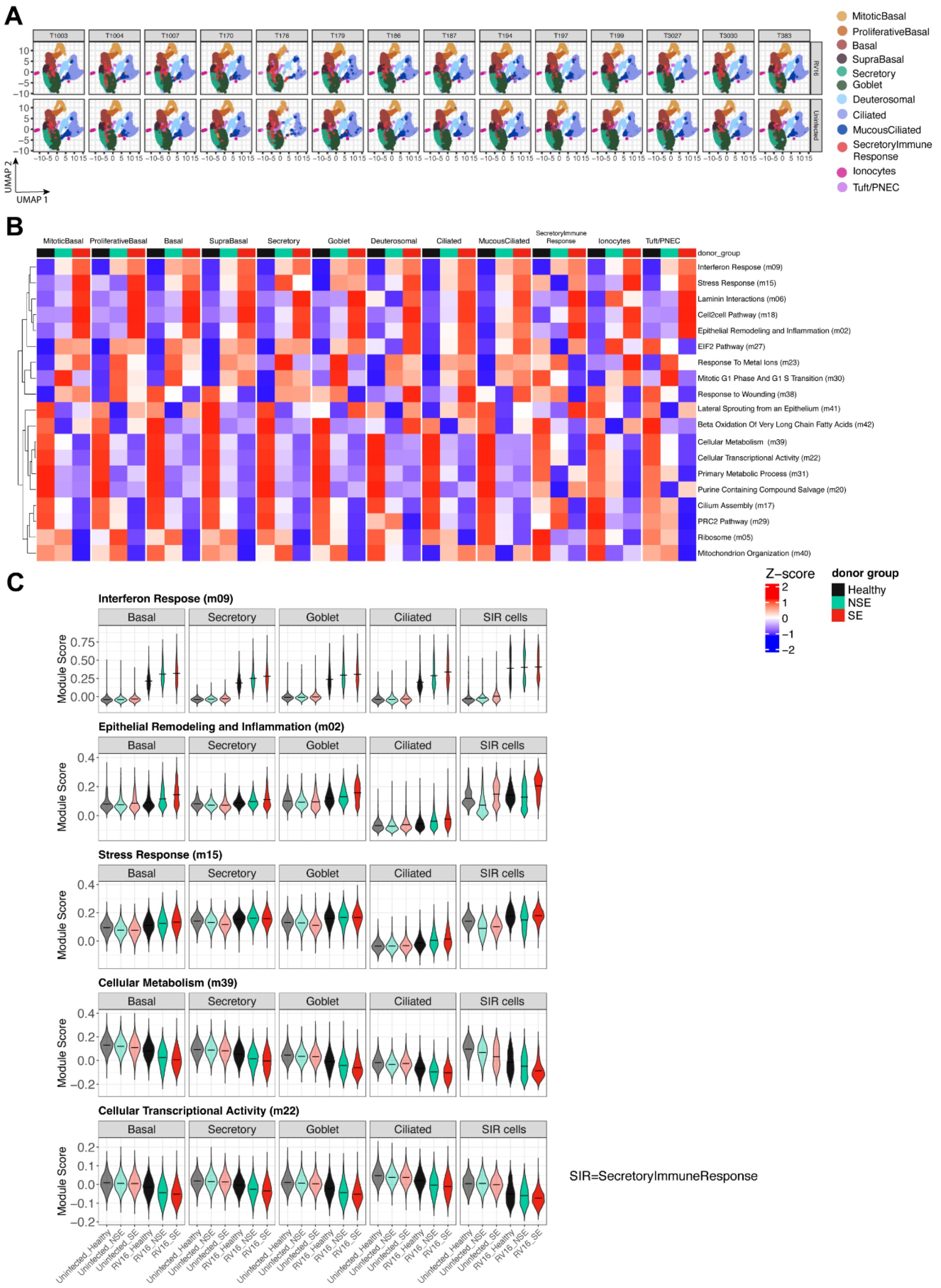

bioRxiv preprint doi: https://doi.org/10.1101/2025.10.19.683298; this version posted October 19, 2025. The copyright holder for this preprint (which was not certified by peer review) is the author/funder, who has granted bioRxiv a license to display the preprint in perpetuity. It is made available under aCC-BY 4.0 International license.

Supplemental Figure 8

A

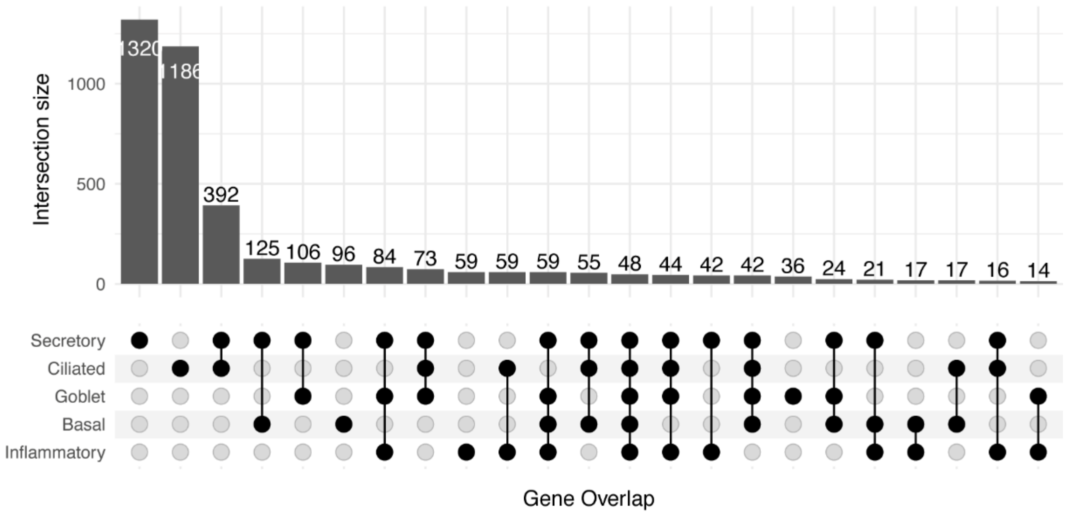

B

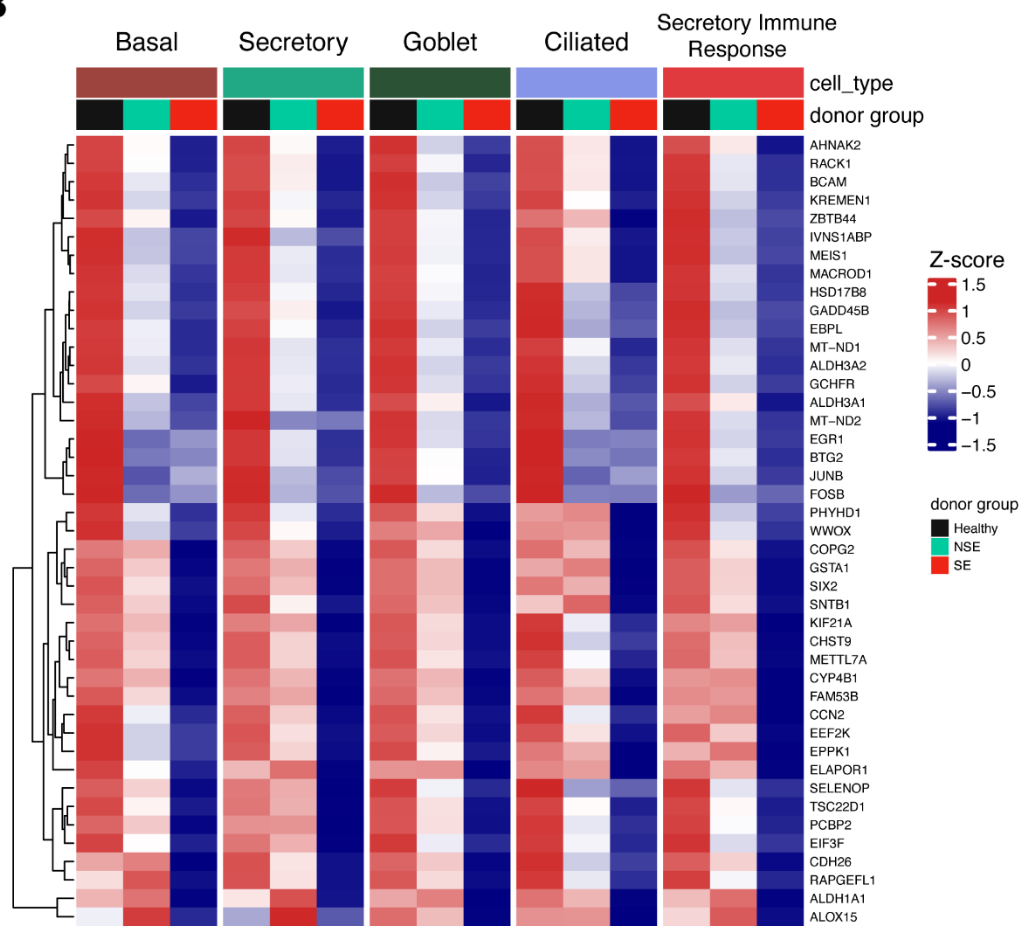

bioRxiv preprint doi: https://doi.org/10.1101/2025.10.19.683298; this version posted October 19, 2025. The copyright holder for this preprint (which was not certified by peer review) is the author/funder, who has granted bioRxiv a license to display the preprint in perpetuity. It is made available under aCC-BY 4.0 International license.

Supplemental Tables

Supplemental Table 1. Generalized Additive Mixed Models (GAMMs) results comparing expression differences over time between healthy and the two exacerbation groups. Table shows the smoothing terms of time and smoothing interaction term for the effect of exacerbation groups and healthy samples over time and shows the average difference by the exacerbation and healthy groups.

Supplemental Table 2. Results of the Linear Model shows the differential expressed modules by viral load over time and subset to Day 2

Supplemental Table 3A: Module annotations based on gene enrichment
Supplemental Table 3B: Table of genes in Modules EnsembleIDs
Supplemental Table 3C: Table of genes in Modules hgnc symbol

Supplemental Table 4. Linear Model results comparing expression at Day 2 between healthy and the two exacerbation groups.

Supplemental Table 5. Linear mixed effects models results to check for mediation effect of viral load

Supplemental Table 6. Linear model comparison of baseline BEC supernatant concentrations by exacerbation group

Supplemental Table 7. Linear Model results comparing expression difference between RV16, IFNb and CXCL10 Treatment groups.

Supplemental Table 8. Linear Model comparison of viral copy number to the module expression.

Supplemental Table 9. Linear mixed effects models results to check for mediation effect of viral load with Treatment group RV16, RV16 + IFNb and RV16 + CXCL10

Supplemental Table 10. Cell Type-Specific Marker Genes. This table lists the top marker genes that distinguish each cell type from the others, as identified through differential expression analysis. These markers were used to define and validate cell type annotations across the dataset.

Supplemental Table 11. Cell Counts by Donor, Condition, and Cell Type. This table provides a breakdown of cell counts stratified by individual donor ID, donor group (healthy controls, NSE asthma, SE asthma), experimental condition (uninfected vs.RV-infected), and annotated cell type.

bioRxiv preprint doi: https://doi.org/10.1101/2025.10.19.683298; this version posted October 19, 2025. The copyright holder for this preprint (which was not certified by peer review) is the author/funder, who has granted bioRxiv a license to display the preprint in perpetuity. It is made available under aCC-BY 4.0 International license.

Supplemental Table 12. CLM Module-Level Estimates in RV-Infected Samples
Contains output from the Cumulative Linked Model (CLM) ordinal regression framework, reporting model estimates for all modules across all cell types within the RV-infected condition.

Supplemental Table 13. CLM Gene-Level Estimates in RV-Infected Samples
Contains output from the Cumulative Linked Model (CLM) ordinal regression framework, reporting model estimates for genes across all cell types within the RV-infected condition.

Supplemental Table 14. Enriched Biological Terms from CLM-Identified Genes
Lists significantly enriched biological pathways and gene ontology terms derived from the set of genes identified as significant in the CLM model under RV-infected conditions.
